# The challenges involved in elucidating the molecular basis of sperm–egg recognition in mammals and approaches to overcome them

**DOI:** 10.1007/s00441-015-2243-3

**Published:** 2015-07-30

**Authors:** Gavin J. Wright, Enrica Bianchi

**Affiliations:** Cell Surface Signalling Laboratory, Wellcome Trust Sanger Institute, Hinxton Cambridge, UK

**Keywords:** Egg, Fertilization, Juno, Izumo1, Membrane protein

## Abstract

Sexual reproduction is used by many different organisms to create a new generation of genetically distinct progeny. Cells originating from separate sexes or mating types segregate their genetic material into haploid gametes which must then recognize and fuse with each other in a process known as fertilization to form a diploid zygote. Despite the central importance of fertilization, we know remarkably little about the molecular mechanisms that are involved in how gametes recognize each other, particularly in mammals, although the proteins that are displayed on their surfaces are almost certainly involved. This paucity of knowledge is largely due to both the unique biological properties of mammalian gametes (sperm and egg) which make them experimentally difficult to manipulate, and the technical challenges of identifying interactions between membrane-embedded cell surface receptor proteins. In this review, we will discuss our current knowledge of animal gamete recognition, highlighting where important contributions to our understanding were made, why particular model systems were helpful, and why progress in mammals has been particularly challenging. We discuss how the development of mammalian in vitro fertilization and targeted gene disruption in mice were important technological advances that triggered progress. We argue that approaches employed to discover novel interactions between cell surface gamete recognition proteins should account for the unusual biochemical properties of membrane proteins and the typically highly transient nature of their interactions. Finally, we describe how these principles were applied to identify Juno as the egg receptor for sperm Izumo1, an interaction that is essential for mammalian fertilization.

## Introduction

Sexual reproduction is a fundamental biological process that is used by many organisms to create progeny that are genetically subtly different from their parents and siblings. Sexual reproduction culminates in fertilization and occurs when two haploid gametes recognize each other and fuse to form a single diploid zygote. The cellular mechanisms used in fertilization vary widely according to the specific lifestyles of different organisms; for example, fertilization can occur internally or externally, and the number of gametes involved can range from the monthly ovulation of a single egg—as is typical in humans—to the release of millions of eggs in some broadcast spawning animals. Despite these differences, there are some commonalities: eggs are typically surrounded by a protective glycoprotein-rich coat that the motile sperm must penetrate, often facilitated by the regulated release of digestive enzymes from an intracellular vesicle in the sperm head called the acrosome. Once the egg investment has been breached, the acrosome-reacted sperm and egg plasma membranes must recognize each other, adhere, and then fuse to form a single, fertilized egg (Okabe 2013). There are good reasons to be interested in the molecules that mediate gamete recognition events, since they are likely to be centrally involved in important biological processes including speciation, self-recognition to avoid inbreeding, and the prevention of polyspermy (Vacquier and Swanson 2011; Evans and Sherman 2013; Kosman and Levitan 2014). Furthermore, because the extracellular regions of receptor proteins are directly accessible to systemically delivered therapeutics, they can be more easily targeted to prevent fertilization, a property that could be exploited for the development of new contraceptives (Kaur and Prabha 2014). Despite the central role of gamete recognition in fertilization, our knowledge of this process at the molecular level is still rather rudimentary, and this is particularly true in mammals. Arguably, the reasons for this paucity of knowledge are two-fold: firstly, due to their unique biology, there are significant experimental limitations in working with mammalian gametes; and secondly, membrane-embedded receptor proteins are difficult to biochemically manipulate. In this review, we will first outline these challenges and describe some of the models and technical advances that have helped address them. We will then discuss some of the biochemical difficulties of identifying extracellular interactions between membrane-embedded receptor proteins and approaches that have been developed to identify this class of protein binding event. Finally, we describe how we have recently applied these principles to discover a sperm–egg receptor pair that is essential for fertilization in mammals.

## The challenges of identifying gamete recognition receptor interactions in mammals

**Fig. 1:**
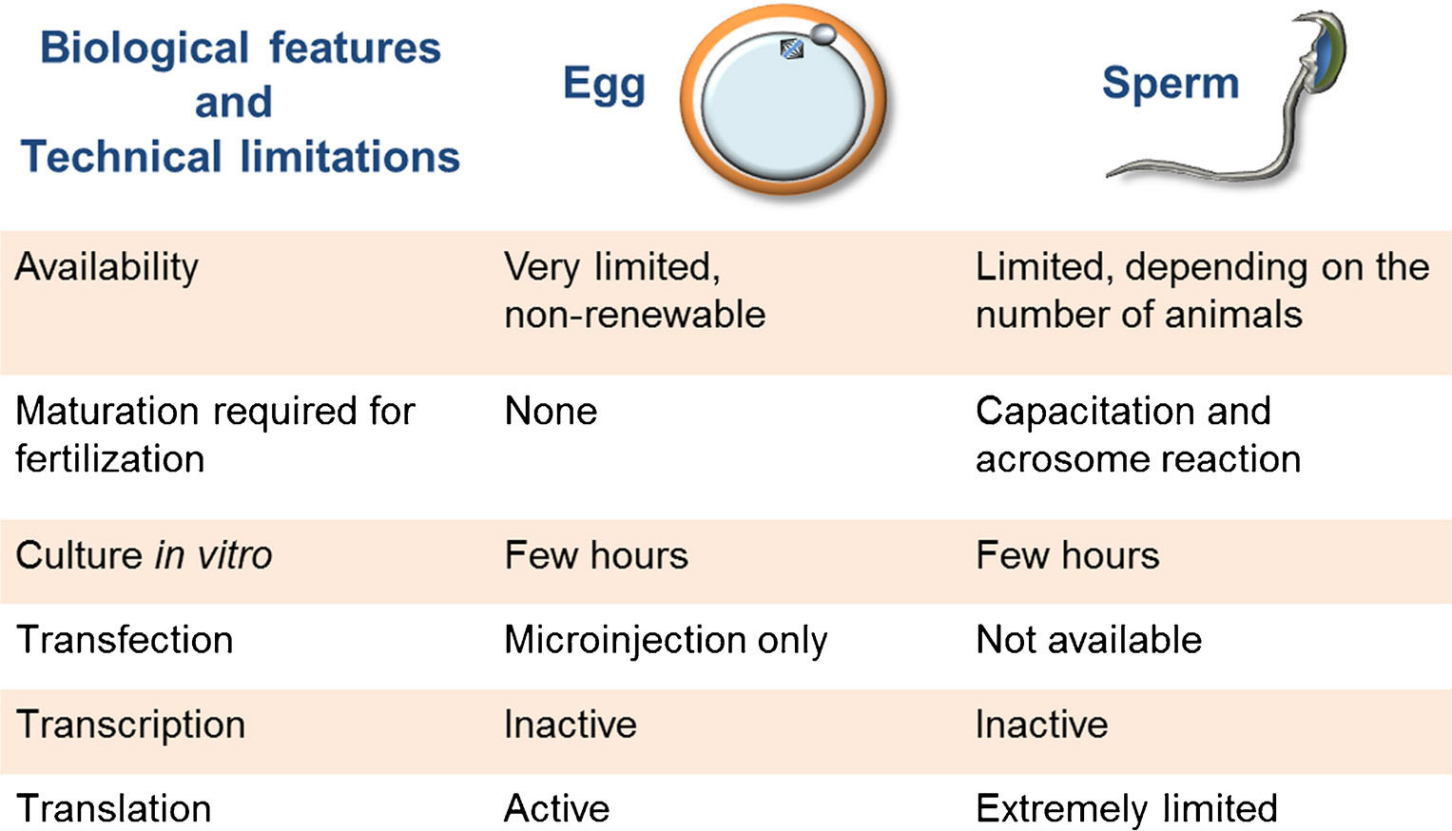
Biological, technical and ethical challenges that have limited progress in the molecular basis of gamete recognition in mammals. Sperm and eggs are terminally differentiated cells whose unique biological properties make investigating the molecular basis of their function difficult. Here, we list some of these properties for both gametes

Mammalian gametes possess unique characteristics that make investigating the molecular basis of their biology challenging (Fig. 1). For example, although sperm can be easily obtained at high purity and in reasonable quantities, by contrast, eggs are a very rare cell type and even highly fecund mammals such as mice normally only produce between 8 and 12 or so oocytes per fertility cycle (Gates 1925). In addition, eggs are not released as discrete cells but are embedded within a cluster of cumulus cells. The difficulty in obtaining large quantities of pure mammalian eggs has therefore largely prevented the application of proteomic approaches such as mass spectrometry to characterize oocyte proteins, although it has been used successfully to identify membrane-associated proteins displayed on the sperm head (Stein et al. 2006). While obtaining fully differentiated mammalian gametes currently requires intact reproductive organs from animals, there has been exciting recent progress in the in vitro differentiation of functional gametes from somatic cells which, in principle, could permit access to limitless quantities of pure gametes (Hayashi et al. 2012; Hayashi and Saitou 2013; Botman and Wyns 2014).

Beyond the scarcity of eggs, terminally differentiated sperm and eggs have the sole purpose of fusing with one another; otherwise, they are destined to die shortly after being released from the reproductive organs. The unusual genetic properties of gametes, and our inability to recapitulate gametogenesis in vitro, have largely prevented the application of technologies that have contributed to advances in other fields. The difficulty of transfecting germ cells during gametogenesis in mammals renders approaches such as RNAi impractical to investigate gene function in sperm, and it has found only limited application in eggs. Similarly, directly injecting in vitro-transcribed mRNAs is a popular technique with *Xenopus* eggs, but with mammalian eggs it is time consuming, and requires expertise in micromanipulation. Finally, mixing human sperm and eggs in vitro is understandably highly regulated by strict ethical guidelines and can therefore only be performed by laboratories that are appropriately accredited. These restrictions make the use of human tissue for basic research purposes impractical, with the consequence that the large number of human reagents that have been assembled by the global biomedical research community over many years, such as antibodies and genomic resources, have not made significant contributions to the study of mammalian gamete recognition. Because of these collective difficulties, the first contributions to our understanding of the molecular basis of gamete recognition were made in other model systems where these challenges could be immediately overcome, and marine invertebrates such as the sea urchin, oyster, abalone and starfish played an important role.

Studying fertilization in marine invertebrates has many advantages. Principally, fertilization is external, and so can be directly observed simply by mixing gametes in sea water. Also, in contrast to mammals, both sperm and eggs can be obtained in large amounts so that even biochemical approaches to identify the molecules involved become feasible. Because of these experimental advantages, progress was possible and the first gamete recognition molecules were discovered in the sea urchin. An abundant acrosomal protein, bindin, was initially purified and characterized from sea urchin (Vacquier and Moy 1977) and later shown to interact with the egg protein EBR1 (Kamei and Glabe 2003). Similarly, the abundance of abalone sperm permitted the purification and characterization of lysin (Swanson and Vacquier 1995) and the subsequent identification of its egg binding partner, VERL (Swanson and Vacquier 1997).

Although the lack of sufficient material may have prevented biochemical approaches in mammals, this restriction does not preclude genetic approaches which have made huge contributions to the study of other biological processes. However, even directed screens to identify fertility genes using forward genetic approaches have largely failed to discover genes involved in mammalian fertilization. At least three large-scale N-ethyl-N-nitrosourea (ENU) mutagenesis screens have been carried out (Hrabe de Angelis et al. 2000; Clark et al. 2004; Sakuraba et al. 2005), and while many sex-specific sterility phenotypes were identified and the causal genetic lesions identified by positional cloning, the function of all identified genes was in germ cell development rather than fertilization itself (Furnes and Schimenti 2007). Similarly, other genetic model organisms, such as yeast, *Drosophila* and *C. elegans*, while they have identified many genes that could be involved, arguably none have directly led to a deeper molecular understanding of mammalian fertilization (Wakimoto et al. 2004; Singson et al. 2008). Instead, the two main technical advances that have significantly contributed to our current molecular understanding of mammalian fertilization have been the development of in vitro fertilization (IVF) using mammalian gametes, and the ability to create targeted gene-deficient mice.

## The development of in vitro fertilization in mammals

Prior to the development of in vitro fertilization methods, there were very few approaches that could be used to study the molecular basis of fertilization; therefore, the elucidation of the conditions needed to capacitate sperm and successfully fertilize eggs in vitro was a genuine breakthrough in the field and quite rightly regarded as a major biomedical success story enabling infertile couples to conceive (Edwards et al. 1970). Beyond these practical medical applications, the development of IVF now provided a reductionist functional assay to begin the process of determining the individual contributions of defined molecules by, for example, adding antibodies to IVF assays and quantifying their effects. Also, together with monoclonal antibody technology, an unbiased systematic “shotgun” approach could be used to select panels of monoclonal antibodies which prevented fertilization and a means to subsequently determine the molecular identity of the components involved (Aitken et al. 1981). This accessible approach led to the identification of many candidate molecules (Table 1) including Fertilin (Primakoff et al. 1987), a heterodimeric protein initially reported to be composed of A Disintegrin And Metalloprotease 1 (Adam1, Fertilin α) and Adam2 (Fertilin β) displayed on the sperm surface; subsequently, it was shown that Adam1 was composed of two isoforms encoded by two genes, *Adam1a* and *Adam1b* (Nishimura et al. 2002). Both Adam1 and Adam2 share similar molecular characteristics including a metalloprotease domain, a disintegrin domain, a cysteine-rich domain, and an EGF-like repeat. The presence of a putative integrin binding site within Fertilin β/Adam2 (Myles et al. 1994) led to the hypothesis that they bound integrins displayed on the oolemma, and experimental support was obtained for this (Almeida et al. 1995).

**Table 1:**
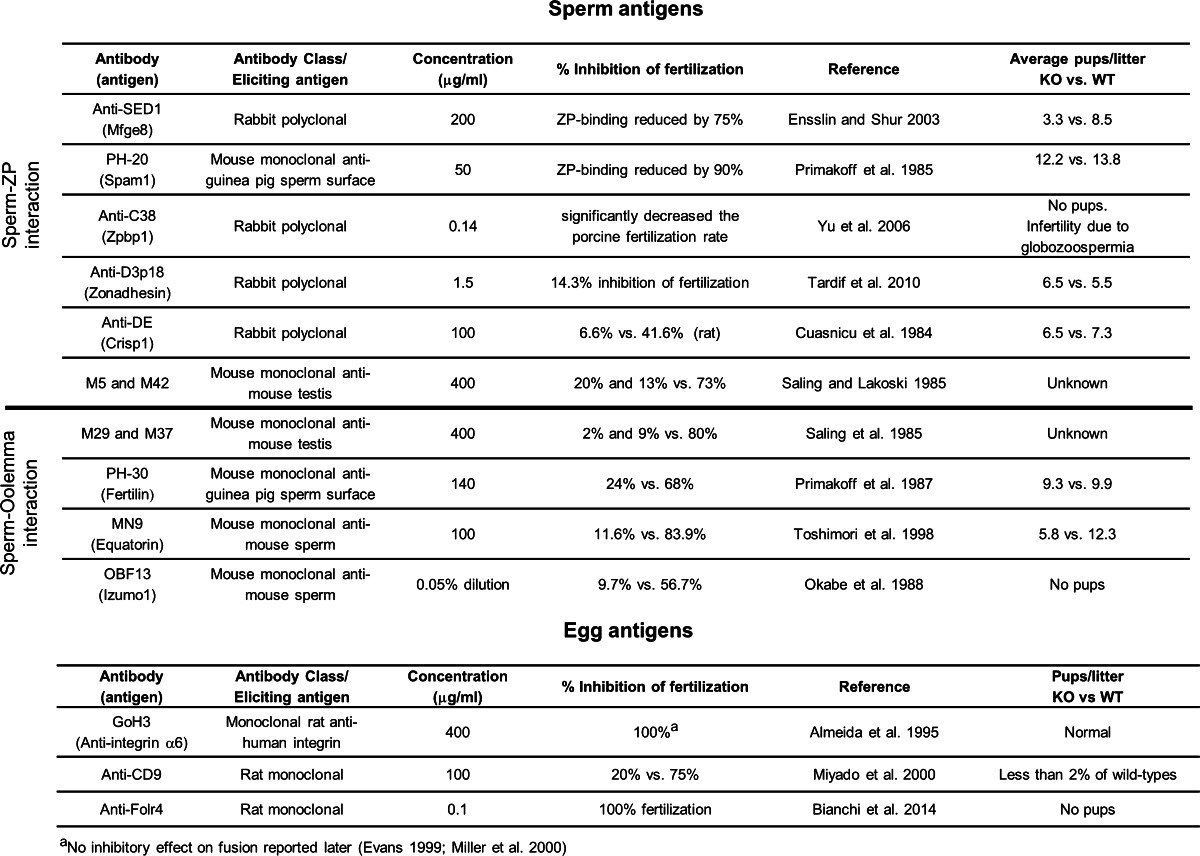
Summary of the effects that antibodies added to IVF assays had on fertilization rate. Antibodies are initially categorised as recognizing egg or sperm antigens, and in the latter case, further subdivided into affecting either the sperm interaction with the zona pellucida (ZP) or oolemma. The antibody class (polyclonal / monoclonal), together with the concentration used to cause the listed effects on fertilization in an IVF assay are shown. Note that in some cases, the antigen recognized by the antibody is not known. The final column compares, where known, the fertility in mice which have a targeted deficiency in the gene encoding the listed antigen. Note that in some cases, there is discordance between the potent inhibitory effect of the antibody in IVF assays, and only a subtle effect on the fertility of gene-deficient mice

^a^No inhibitory effect on fusion reported later (Evans 1999; Miller et al. 2000)

The strategy of raising monoclonal antibodies to sperm antigens that blocked fertilization in vitro resulted in the selection of OBF13 (Okabe et al. 1988). The antigen recognized by this antibody was found to be a cell surface protein belonging to the immunoglobulin superfamily and was named Izumo1, after a Japanese marriage shrine. Izumo1 is displayed on the surface of acrosome-reacted sperm and was eventually shown to be essential for fertilization (Inoue et al. 2005). Finally, another important contribution made possible by the development of IVF was to show that zona pellucida-free eggs could be rendered infertile by treating them with the enzyme phosphatidylinositol phospholipase C (PIPLC), which removes surface proteins that are tethered to the membrane through a glycosylphosphatidyl inositol (GPI) lipid modification. This suggested that there were one or more GPI-anchored receptor proteins on the egg that were necessary for sperm recognition or fusion (Coonrod et al. 1999).

The development of IVF therefore directly contributed to the identification of several molecules that were involved in gamete recognition and the beginnings of a molecular model began to form. This initial molecular framework, however, was about to change with the development of a new technology: the ability to create gene-deficient mice.

## Targeted gene-deficient mice and their role in the molecular understanding of mammalian fertilization

In the 1990s, the ability to indefinitely culture embryonic stem cells in a pluripotent state and select genome targeting events led to the ability to create gene-deficient “knockout” mice (Kuehn et al. 1987; Thomas and Capecchi 1987). This technology permitted the in vivo relevance of the molecules that had been implicated in mammalian gamete recognition to be directly tested, something that cannot generally be performed in marine invertebrates. Unexpectedly, targeted gene knockout for several of the candidate molecules did not cause infertility, but rather the mice were found to be just subfertile, or entirely fertile (Table 1). For example, sperm that lacked Adam1b, which forms an Adam1b/Adam2 heterodimer at the sperm surface (Kim et al. 2003), retained their ability to fertilize eggs (Kim et al. 2006). Mice deficient in Adam2 showed a strong reduction of sperm ability to bind the zona pellucida and the oolemma (Cho et al. 1998), but while the fertility phenotype was initially thought to be due to the lack of a ligand on sperm, it was subsequently shown to be due to the disruption of the Adam1a/Adam2 heterodimer which is exclusively expressed in the testes and is involved in the localization of another Adam family member on the sperm surface, Adam3 (Nishimura et al. 2004; Kim et al. 2006). The roles of the putative Adam binding partners on the egg, the integrins, were also investigated. Specifically, the integrin α6β1 is expressed on the egg membrane and both an antibody against integrin α6, and a peptide analog of the fertilin integrin ligand domain, inhibited sperm–egg fusion (Almeida et al. 1995). Again, the use of gene-deficient mice showed that the emerging model for mammalian gamete recognition was incomplete since mice lacking integrin α6 were fertile (Miller et al. 2000). Thereafter, targeted gene deletion in mice became the ‘gold standard’ in assessing the functional relevance of the protein of interest in vivo.

As more and more gene-deficient mice were created, and because fertility is one unequivocal phenotype that is almost always tested when establishing a breeding colony of mice, there came the serendipitous discovery that Cd9 was required for female fertility. Female *Cd9*-deficient mice are severely subfertile due to the inability of their eggs to fuse with normal sperm, perhaps a surprising finding given that Cd9 is expressed on many cell types, and had been previously implicated in several diverse functions including cell adhesion, motility, proliferation, differentiation, and signal transduction (Maecker et al. 1997). Cd9 is an integral cell surface membrane protein and a member of the “tetraspanin” family, so-called because they possess four membrane-spanning helices. The mechanism by which Cd9 functions is still not completely understood, with most evidence pointing to an architectural or organizational role for other proteins embedded within the membrane (Jegou et al. 2011; Chalbi et al. 2014). There is evidence that Cd9 modifies the curvature of the microvilli found on the oolemma, which has led to the suggestion that it might influence sperm–egg membrane fusion (Runge et al. 2007). Interestingly, female mice that are doubly deficient in both Cd9 and another member of the tetraspanin family, Cd81, are completely infertile due to the inability of sperm to fertilize the double knockout eggs, suggesting that there is some redundancy in their function in the egg membrane (Rubinstein et al. 2006). Lastly, gene-deficient mice were used to genetically confirm the requirement of an essential GPI-linked cell surface receptor on the egg membrane by conditionally deleting the enzyme *Pig-a*, an enzyme necessary for GPI-anchor biosynthesis in oocytes (Alfieri et al. 2003).

The use of gene-deficient mice was again central in establishing the function of Izumo1 as the first essential sperm cell surface protein in fertilization (Inoue et al. 2005). Izumo1 was purified by immunoprecipitation using the OBF13 monoclonal antibody from sperm extracts, and the derived peptides matched a previously uncharacterized cDNA sequence which encoded a type I membrane protein with an extracellular immunoglobulin (Ig) superfamily domain and a short cytoplasmic C-terminal tail (Inoue et al. 2005). Interestingly, Ig-like domains show structural similarity to C2 domains, which are often found in proteins that mediate Ca^2+^-dependent protein–protein and protein–membrane interactions (Rizo and Sudhof 1998). Initially, the localization of Izumo1 on the equatorial region of the acrosome-reacted sperm head and the presence of an Ig-like domain suggested the idea that it might be the molecule responsible for sperm–egg fusion. The finding that Izumo1-deficient sperm could bind, but not fuse with, zona-free oocytes (Inoue et al. 2005) led to the view that Izumo1 might function as a unidirectional fusogen, a model that was undoubtedly influenced by the emerging work on viral–host cell fusion. Since then, different laboratories have tested this hypothesis using cell–cell and cell–egg fusion assays which concluded that Izumo1 did not function as an isolated fusogen, but rather formed part of an essential adhesion complex with the egg (Inoue et al. 2013; Bianchi et al. 2014). The realization that there were essential sperm–egg cell surface molecular recognition events led to the search for specific egg binding partners of Izumo1; however, identifying novel extracellular receptor interactions once again presented a set of experimental challenges.

## Biochemical challenges in the detection of novel low affinity extracellular receptor interactions

The investigation of the molecular basis of gamete recognition in mammals is additionally complicated by the biochemical intractability of working with membrane-embedded receptor proteins and their interactions. Membrane proteins are amphipathic; that is, on the same molecule, there is a stretch of hydrophobic membrane-spanning amino acids as well as typically containing very hydrophilic glycans - these different physiochemical properties make membrane proteins hard to solubilize in solvents that retain their native conformation (Whitelegge 2013). In addition, because their ectodomains are exposed to an oxidizing extracellular environment, they contain disulfide bonds which are necessary to ensure the proteins fold correctly, and these structurally important modifications are sometimes not faithfully added in many prokaryotic or cell free heterologous expression systems. Although there are an increasing number of bespoke methods to characterize cell surface receptor proteins (Durr et al. 2004; Nunomura et al. 2005; Zhou et al. 2007; Wollscheid et al. 2009), and rapid improvements in the sensitivity of mass spectrometry methods suggest that it may soon be possible to routinely profile these proteins even from very small amounts of biological material. However, previous efforts to characterize the proteins expressed by mouse oocytes did not identify many, if any, cell surface proteins (Zhang et al. 2009).

Beyond the difficulties in biochemically manipulating and identifying membrane proteins, detecting the extracellular intercellular binding reactions that they mediate is also difficult because, when measured, these interactions are usually found to be highly transient, in many cases often having half-lives of just fractions of a second (van der Merwe and Barclay 1994). These interactions have evolved to be so weak because these proteins are unlikely to interact as discrete pairs; rather, whole Velcro-like arrays of many tens, hundreds or thousands of molecules presented on opposing membranes would associate at any one time (Wright 2009). Collectively, many weak interactions additively ensure sufficient intercellular adhesion, and yet the fleeting and dynamic nature of the component parts ensures cells are able to separate, if necessary. The requirement for highly selective and yet reversible cellular recognition processes is well appreciated by immunologists, puzzling over the problem of how rare circulating antigen-specific T-cells must scan the surface of antigen presenting cells within the lymph nodes. The transient nature of extracellular protein interactions has been observed in many different biological contexts beyond the immune system ranging from myoblast fusion in zebrafish (Powell and Wright 2011) to the recognition of the erythrocyte by the blood stage of the malaria parasite (Crosnier et al. 2011), suggesting that it is a universal feature of this class of interactions. To circumvent these challenges, most approaches rely on producing the ectodomains of cell surface receptors using mammalian or insect cell expression systems so that they are correctly folded, and then adding a recombinant protein tag that multimerises the protein so as to achieve a gain in overall binding avidity (Wright 2009). These approaches range from using fluorescent beads (Brown et al. 1995), using Fc-tags (Chamow and Ashkenazi 1996), clustering around tetrameric streptavidin (McMichael and O’Callaghan 1998), or other tags (Wright 2009). When detecting these interactions, it is essential to build these factors into the design of the experiment.

The approach adopted in our own laboratory has been to use a short peptide sequence from a cartilage protein called the cartilage oligomeric matrix protein (COMP), which has the intrinsic property of forming pentamers (Malashkevich et al. 1996), thereby increasing the local concentration of the expressed ectodomains. This clustering increases the overall binding avidity such that interactions which have monomeric half-lives of just a few fractions of a second can be artificially increased to many tens of minutes or hours thus permitting their detection (Voulgaraki et al. 2005). This technology has been applied in an assay called AVEXIS (for avidity-based extracellular interaction screen) in which the ectodomains of membrane proteins are expressed either as monomeric ‘baits’ or enzyme-tagged pentameric ‘preys’. The assay works by detecting direct protein interactions with the bait protein immobilized in microtitre plates in an ELISA-style format (Bushell et al. 2008; Kerr and Wright 2012), or glass slides (Sun et al. 2012, 2015), that can be scaled to systematically test many thousands of interactions in parallel. Similar screening assays have been developed by others (Wojtowicz et al. 2007; Ozkan et al. 2013), but all share the two core principles: that the ectodomains are expressed in eukaryotic cells to ensure correct folding of the ectodomain region, and binding avidities are increased using multimerising tags. The application of these approaches is likely to make important contributions to elucidating the molecular basis of cellular recognition process in many different biological contexts, including mammalian gamete recognition.

## Identification of Juno, the Izumo1 receptor

**Fig. 2:**
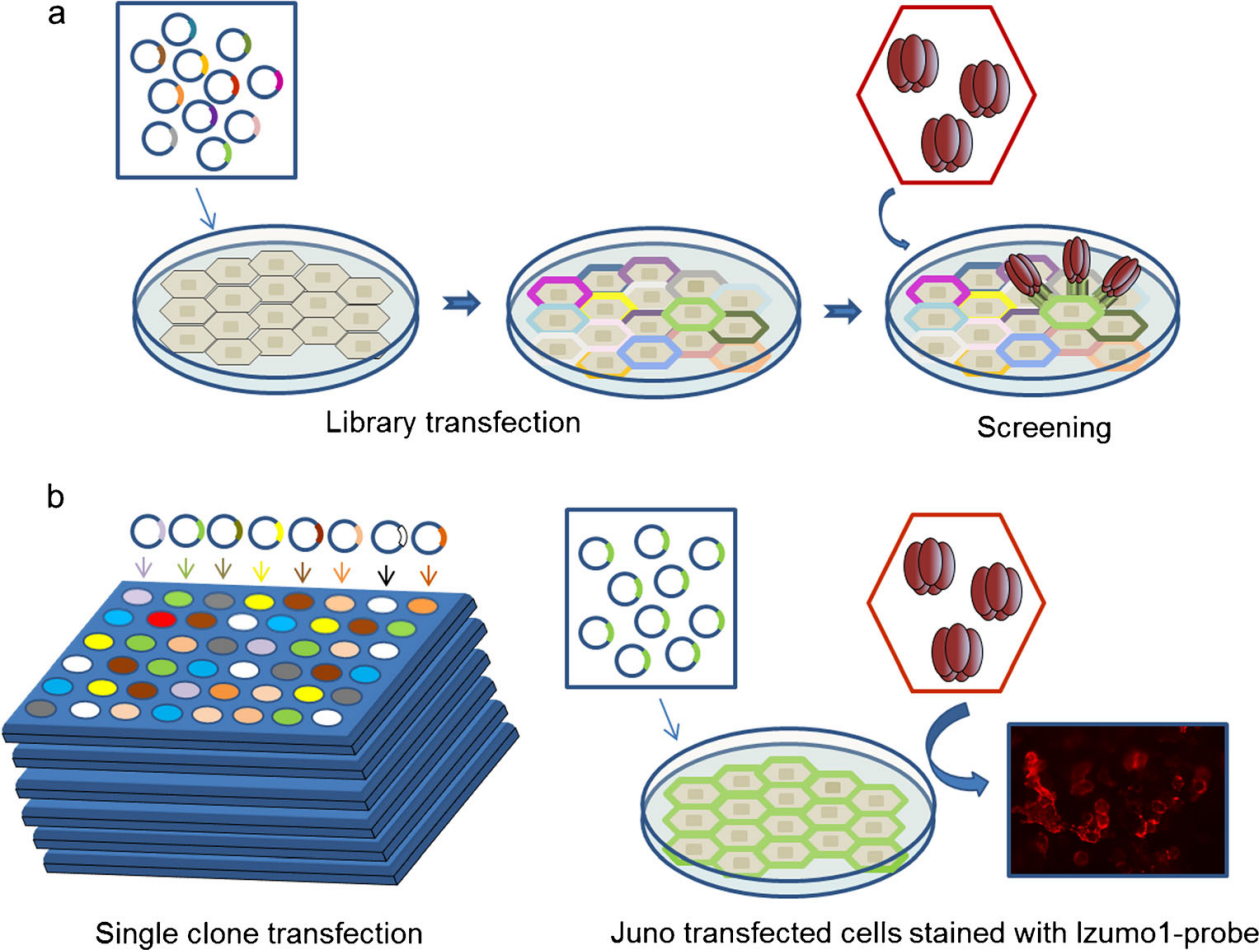
The use of a highly avid ectodomain probe and an expression cloning approach to identify the Izumo1-Juno interaction. **a** Pools of plasmid clones from a mouse oocyte cDNA expression library were transiently transfected into adherent HEK293T cells. A highly avid (pentameric) tagged Izumo1 ectodomain (*brown ovals*) binding probe was used to identify plasmid pools that contained an Izumo1 binding protein by positively staining the transfected cells. **b** Individual plasmids within the positive pools were tested for their ability to confer gain of Izumo1 binding. Identified plasmids were then sequenced to reveal the identity of the Izumo1 binding protein which, in all cases, was Juno

With the expectation that the interaction affinities between sperm–egg recognition receptors would be weak, we were able to successfully identify the egg receptor for the sperm Izumo1 protein (Bianchi et al. 2014). The entire ectodomain of Izumo1 was produced as a highly avid soluble recombinant pentamerized probe that, after showing it could bind to the oolemma, was used to screen a mouse oocyte cDNA expression library for binding partners using an expression cloning technique (Aruffo and Seed 1987; Caterina et al. 1997). Briefly, adherent HEK293T cells were transfected with pools of clones from the cDNA library and screened for their ability to bind the Izumo1 probe (Fig. 2). Pools of clones that were able to confer the ability to bind Izumo1 were iteratively resolved until just single clones were obtained. Sequencing revealed these clones encoded a gene known as Folate Receptor 4, which, because it is unable to bind folate, and since *Juno*-deficient female mice were infertile, we proposed to rename it “Juno” after the Roman goddess of marriage and fertility. As expected, the interaction affinity between Juno and Izumo1 is very weak, with a monomeric half-life of approximately just half a second. Reassuringly, Juno was demonstrated to be tethered to the egg surface by a GPI anchor consistent with the previous findings of an essential GPI-linked protein required for egg fertility (Coonrod et al. 1999; Alfieri et al. 2003). We showed that Izumo1–Juno binding was conserved across several mammalian species, including humans, and that human Izumo1 could interact with hamster Juno (Bianchi and Wright 2015), providing a molecular explanation for the ability of human sperm to fuse with hamster eggs. Finally, the rapid shedding of Juno within vesicles shortly after fertilization provided a plausible molecular explanation for the membrane block to polyspermy, the process by which eggs become refractory to further sperm fusion events once fertilized in order to prevent the generation of polyploid embryos.

## Future perspectives and concluding remarks

The cells in our body develop special characteristics depending on the tissues they form: they possess different shapes and sizes and produce substances as different as hormones, enzymes and bones. Among the ∼200 different types of cells present in a human body only the fusion of two different germ cells, the sperm and the egg, are able to create a new organism to ensure the reproduction of the species. This process is clearly essential but, because of the technical challenges we have outlined here, we have struggled to identify the molecular mechanisms involved. Perhaps because of these difficulties and some initial false leads (Almeida et al. 1995; Cho et al. 1998), scientists have been wary about taking the risk of embarking on the search for molecules involved in sperm–egg interactions. So far, Izumo1 and Juno are the only pair of proteins essential for fertilization in mammals, but, while essential, they are not sufficient for cellular fusion; therefore, it is highly likely that other proteins will be involved. The continual development of new approaches is helping to circumvent some of the technical barriers we have outlined here, and will permit further progress in our molecular understanding of mammalian gamete recognition. Perhaps surprisingly, we have not yet developed a simple diagnostic method to evaluate the fertilizing ability of sperm and eggs beyond a basic morphological description, and that these advances would benefit many applications that rely on in vitro fertilization, such as assisted human fertility and livestock production. The involvement of Izumo1 and Juno in cases of human infertility has not yet been fully investigated, nor has their potential use for molecular contraception. Much, therefore, is still left to be discovered before a comprehensive view of fertilization is achieved.
